# Measuring with quality: the example of person-centred care

**DOI:** 10.1177/13558196211054278

**Published:** 2022-03-02

**Authors:** Alan Cribb, Thomas Woodcock

**Affiliations:** 1 Professor of Bioethics and Education, Centre for Public Policy Research, 121212 King’s College London, UK; 2 Improvement Science Fellow, Faculty of Medicine, 4957 Imperial College London, UK

**Keywords:** outcome measurement, qualitative methods, person-centred care

## Abstract

Qualitative data analysis should be embedded in routine health service measurement, management and organizational practices. The rigorous use of such analyses should become an institutional norm, comparable to the routine use of quantitative data. Our case is intended to have general relevance, but we develop it by reference to person-centred care and patient-centred outcome measures (PCOMs). The increased use of qualitative data analysis of individualized PCOMs is a crucial complementary counterweight to steps towards the standardization of PCOMs. More broadly, our argument is that health care organizations cannot make confident judgements about whether they are offering appropriate care without collecting qualitative data on what matters to individual patients. Introducing properly supported and conducted qualitative data analyses is important in its own right, and also helps underpin the validity and usefulness of quantitative measurement.

## Introduction

The use of qualitative data analysis should become an institutional norm in health services, comparable to the routine use of quantitative data analysis. Health care organizations cannot make confident judgements about whether they are offering appropriate care without paying attention to qualitative data on what matters to individual patients. An expansion of properly supported and conducted qualitative data analyses would help underpin the validity and usefulness of quantitative measurement, which could otherwise be granted undue credibility, with damaging consequences.^1^ Although we make our case with reference to person-centred care, we see it as having general relevance.

### Person-centred care

Person-centredness is a multi-dimensional and contested concept which we cannot do justice to here.^2–6^ But we should start by acknowledging its complexity and the breadth of its potential applicability before focussing down on the relatively narrow example of person-centred outcome measures (PCOMs). Person-centredness is sometimes used interchangeably with patient-centredness, but the former, which we will mainly adopt, has less restricted connotations. In particular, it indicates both the broader identities and social contexts of people beyond health care encounters and the underpinning ethical rationale of ‘centring’ care around people as autonomous agents, and not as passive recipients of care.^6–7^ Person-centredness is invoked within a range of discourses and at micro-, meso- and macro-policy levels nationally and internationally. For example, it is found in a range of policy discourses that emphasize consumerist, deliberative and/or participatory elements^8^ – which, separately and in combination, can give person-centred thinking contrasting ideological emphases. Similarly, Pluut,^9^ looking more narrowly at the literature on patient-centredness within medical encounters, draws out three overlapping but distinct discourses – around ‘caring’, ‘empowerment’ and ‘responsiveness to individual differences and contexts’ – which can produce quite different inflections of the concept.

These variations are also reflected and amplified with different disciplinary and policy contexts. For example, within discussions of clinical quality, person-centredness (often ‘patient-centredness’) is typically treated as one of a number of axes for quality improvement. For example, it is one of six dimensions within the influential Institute of Medicine account of quality,^10^ However, within discussions of health system reform or transformation (or system-level quality) person-centredness can be used a shorthand for an overarching vision or axis of service reconfiguration.^11^ Nonetheless, despite this diversity, there are a few key ideas that underpin the concept, notably that person-centredness is usually contrasted with models that are seen as too narrow (for example, disease-centred or biomedically-centred health care) and involves treating service users as people, and respecting and responding to their values and preferences.^12^ Given these critically important and increasingly recognized principles, person-centredness has come to be treated globally as both a key facet of good quality care and as a health systems policy priority.^13^

### Measuring person-centred care

Although there is considerable consensus about the fundamental importance of person-centred approaches to health care, there is no equivalent consensus about how person-centred care can or should be measured. These two facts do not sit comfortably together. We propose that the routine adoption of qualitative data analysis by health services can help to address this conundrum.

Researchers and health service organizations have built up a considerable body of expertise in developing measures for patients’ perceptions and experience of care and, in some cases, for embedding these into routine use.^6^ Many of these measures relate to processes and climates of care – thus they are relevant to assessing person-centredness at system or service levels. Both quantitative and qualitative data collection and analyses have relevance across this whole terrain. However, for our illustrative example, we will only focus on those measures which look, more narrowly, at service outcomes as assessed by patients – patient-reported or patient-centred outcome measures (PROMs or PCOMs). These kinds of measures have risen to international prominence because they can provide insights into outcomes that pose difficulties for, or often are literally inaccessible to, staff assessment. This is because they can, for example, capture experiences and meanings that are ‘internal states’ or that relate to life beyond health care encounters.^14^

Here we will just stress one justification that might be offered for the importance of PCOMs, arguably a central one. Specifically, it is very difficult to claim that services are valuable (or effective, safe and so on) unless they are experienced as valuable (or effective, safe and so on) by the people they are designed to serve. This crucial insight – discussed by Mulley and colleagues as addressing the problem of ‘preference misdiagnosis’ – has very significant implications for planning and managing services.^15^ For this reason, the same impetus that has led to the rise in salience in person-centred care has produced pressure to find better means – that is, more person-responsive means – of evaluating health services. This is often translated into a measurement challenge: *Can we find ways of measuring the things that actually matter to patients and can we try to ensure we are not measuring the wrong things*?^16^

PCOMs capture outcomes that are valuable to service users, and thereby arguably provide a key to quality, including, as advocates of person-centred care might suggest, to the ‘real’ effectiveness of services. Understood in this sense person-centredness is not merely one dimension of quality^10^ but can be seen as a lens through which the other dimensions might be better discerned and appraised.

PCOMs can be designed in a range of ways. They are all – by definition – informed by patient perspectives. But they can be constructed in ways that are themselves more or less person-centred, not all of which are equally suited to the development of metrics. There are two relevant dimensions here – first, the degree to which PCOMs should be ‘patient-generated’, that is, defined by service users (as opposed to merely being informed by them) and, second, the degree to which PCOMs are individual-specific (as compared with being designed to fit groups of patients on the same pathways or more broadly). More standardized PCOMs lend themselves to various degrees of quantification. By contrast, the use of qualitative data is particularly suited to greater individualization.

It is, of course, quite commonplace for people interested in care quality to draw on qualitative data. The Institute for Healthcare Improvement, for example, recommends as one of its tips for effective measurement: ‘In addition to collecting quantitative data, be sure to collect qualitative data.^17^ (p 1) There is also now a body of scholarship on person-centred research and on person-centred data collection methods such as interviews, photo elicitation and video-reflexive ethnography.^18,19^ If measures are to embody the most full-bodied person-centredness they will capture ‘what matters’ to individuals and do so in terms that individuals personally identify with – what differences have any interventions made to their lives and how far have their interactions with services helped them to meet the purposes they had hoped for and/or expected? NHS England has, for example, run pilots to experiment with PCOMs. In some of these, individuals were able to define, assess and monitor – and thereby maintain – an ongoing and shared record of their own (self-defined) valued outcomes from treatments.^20^

The possibility of flexible and responsive outcome data reflects something very important. But it also highlights a difficult challenge. Is it possible to have PCOMs that have both a high level of individualized responsiveness and a high level of service utility? The more emphasis we place on individualized and self-defined measures, the less general relevance these measures seem to have as they become increasingly less commensurable with one another and more difficult to aggregate. For that reason, what we have called full-bodied PCOMs tend not to be used on a large scale but are rather seen as more suitable for clinical consultations and care planning.^6^ It is generally assumed that they have less utility for other important ends – such as the comparisons between cases, sites and over time – that can be used to guide service planning and evaluation. For these latter purposes, more impersonal measures seem preferable to personalized measures. However, both more personalized and less personalized PCOMs have crucial relevance. The former enable us to understand the full set of interactions between services and lives and to strengthen the individual tailoring of services. Without them we are hampered in knowing how far organizations are offering appropriate care.^21^ The latter enable us to understand the broad effectiveness of services across relevant sub-populations, over time and in comparisons between providers.

It is possible to design overarching measures that combine these different concerns, but attaining compatibility requires sacrificing some sensitivity. For example, there are measures that go some way towards capturing personalized care success whilst allowing a degree of aggregation – for example, a goal-setting and achievement type approach which can both attach a weight to how far specific individuals have been able to attain the treatment goals they have set, and also aggregate the attainment achieved by samples of patients.^22^ Of course, these do not go all the way to capture completely personalized outcomes, and require very careful implementation in order to avoid a collapse of validity. But they represent one plausible attempt to combine personalization and standardization.

It makes sense for services that are accountable to communities (and often to collective funders such as taxpayers) to be able to aggregate and compare, to assure or improve quality, and hence to place considerable emphasis on less personalized outcome indicators. Indeed, the demands of comparability and quantitative rigour push us towards increasing standardization of measurements and metrology to underpin validity.^14,23,24^ Nonetheless, the good reasons to collect more personalized outcomes also remain. How can services make use of these if they cannot be numerically aggregated or compared? The option we are advocating is to use qualitative data analysis techniques to synthesize them together and present them in a way that reflects their complexity. Qualitative data analysis is expressly concerned with the synthesizing of data – including relatively open-ended and ‘soft’ data – that contains non-commensurable elements. This is not the same as converting individualized PCOMs into more generalized measures (which loses some of their distinctive value), but rather – if done properly – it seeks to preserve something of the richness, depth, diversity and ‘feel’ of the data, including giving due prominence to data that is discrepant, unexpected and perhaps even unsettling in some respects. In practice, what we are suggesting would involve system leaders, managers or practitioners viewing and discussing generalized PCOM measures alongside short qualitative syntheses of more individualized data. If done well, the two kinds of evaluations strengthen one another – the individualized voices highlighting both the broader significance and limitations of the measures, and the measures providing a counterbalance to the risk of over-sensitivity to any particular narrative.

### Adding quality to quantity

Devising suitable measures is both practically and ethically necessary to running services responsibly and defensibly. At the same time, there is an ongoing cultural conversation about the potentially distorting effect of high stakes measures, performance management and audit cultures,^1,25–27^ as popularized through reference to ‘Goodhart’s law’ cited as: ‘When a measure becomes a target, it ceases to be a good measure.^28^ (p 308)’

Quantitative measurement operates as a means to an end and not an end in itself. The underlying purpose is for service providers to have ‘ways of knowing’ the actual or potential characteristics of their services, including, and especially, their effects on people’s lives. The construction and uses of measures can only be defended to the extent that they – directly or indirectly – serve this purpose. Here, as generally, there is no inherent hierarchy between quantitative and qualitative ways of knowing – everything depends on what is being done with them and how well it is being done.

The rigour and usefulness of quantitative measures themselves depend upon them being informed by, and seen in the context of, qualitative ways of knowing. This is something that is widely recognized and accepted in many contexts, not least as part of normal business within social research. But this is not sufficiently acted upon in the running of health care institutions. It is striking that a *BMJ* article providing advice on ‘using data for improvement’ concludes a section on the potential for qualitative data analysis by saying: ‘If you want to try this, see if you can find someone in your organisation with qualitative data analysis skills.^29^ (p 1)’ It is difficult to imagine the same being said for quantitative analysis.

The crucial role of qualitative data can be illustrated through a simple example. If 97% of patients give a service a ‘good’ (or better) score on some measurement tool then what should we make of that? Even assuming that the tool has been validated and is being applied correctly, there are still several important further issues to be addressed. First, when can we assume that the validation remains sufficient as times and contexts change? Second, how far does the tool tell us about what matters most to patients, as opposed to what is important from a system point of view? Third, what should we conclude about the 3% of people who score the service as less than good – are they missing something or are their needs or preferences just different? The risks of ignoring this minority of respondents are potentially very high. For example, doing so may obscure adverse events which are crucial for the monitoring of patient safety. Alternatively, it could mean failing to detect cultural or other variations within patient cohorts, such that services fail to be adequately responsive to diversity or equitable. This latter issue could also have wider international implications if it turns out that tools developed within one national or cultural frame of reference do not have equal applicability in other contexts and need significant adaptation. Fortunately, some of these hazards are avoided in practice because managers and practitioners already rely on numerous sources of qualitative or ‘soft’ intelligence available to them, including through their own routine experiences, observations and conversations.^30^ However, we wish to underline the much greater potential for more systematic uses of qualitative data.

Each of the three questions summarized above can be addressed by the judicious use of qualitative methods. These can provide insights into how the social contexts of care might evolve or vary, about whether tools sufficiently reflect the agendas of a diverse range of service users and about apparently discrepant cases. In short quantitative measurements are much more trustworthy and useful when they are read and interpreted alongside qualitative ways of knowing.

Our proposal depends crucially, of course, on paying attention to qualitative rigour. There is considerably more scope for the canons of qualitative rigour – extensively elaborated and debated within the social sciences literature – to be adopted and applied within health services in a manner that is comparable to the existing use of quantitative analyses. There is now an extensive body of work on what counts as good quality qualitative work.^31^ This includes some measure of debate about how far the relevant criteria of rigour in qualitative methods loosely correspond with or are distinct from those that apply in quantitative methods.^32^ In broad terms, rigour in quantitative research seeks ‘objectivity’ by eliminating bias, whereas qualitative rigour involves systematically acknowledging the strengths and limitations of the data sets and data analyses used. Both approaches are centred on critical scrutiny, and each has relative advantages and weaknesses.

Methodological scholarship within qualitative research includes regular debate about achieving and refining rigour.^33^ But within these exchanges there is a considerable measure of consensus about the basis of establishing ‘trustworthiness’. This includes paying attention to the clarity and auditability of context, sampling, data collection and analysis; the value of respondent validation, triangulation, drawing on rich or ‘thick’ data and on multiple voices or perspectives in a data set; and the importance of ‘reflexivity’ (systematically acknowledging and reflecting on the potential effects of the researcher(s) on the research process and analysis).^34,35^ Qualitative research papers will often have sections that highlight and critically defend the combination of approaches to rigour deployed within the relevant study,^36,37^ and it is widely understood that this involves an element of flexibility and responsiveness to the study aims and design rather than applying a fixed template.^38^ Such flexibility may be needed, for example, to adapt to the challenges of studying minority ethnic populations.^39^ There are many health service researchers who have capabilities in this area.

In summary, both quantitative and qualitative data collection and analysis methods can be used well or badly separately or in combination. However, there are arguably three key contrasts worth highlighting. First, relative to quantitative methods, there is much less connection between the day-to-day use of qualitative ways of knowing within health systems and the expertise available. Second, although qualitative investigations are used, for the most part this qualitative work seems to be done on a smaller scale and without national frameworks of expectation and support. Exceptions include after-the-event, sometimes very intensive, investigations when things go (extremely) wrong. Third, the smaller scale qualitative work that is available within institutions is often given less prominence and granted less credence than quantitative measures by authoritative agents involved in routine institutional decision-making (for example, by boards and senior managers).

These three factors are probably connected. The reason why qualitative approaches are both less widely institutionalized and treated as less authoritative may well reflect the lower degree of literacy about, and ‘translation’ of, models of rigour from the qualitative research methods literature. If this translation work could be progressed in practical ways – which is what we are advocating here – then the profile, credibility and routine usage of qualitative ways of knowing should rise further.

There are, no doubt, some good reasons why within health systems quantitative measures have more institutional currency than qualitative data analyses. Quantitative measures ‘take up less space’ (that is, they can be presented and read relatively quickly) and can be stored, transmitted and computed very efficiently (which makes them extremely useful for the monitoring and learning systems enabled by IT). But, to reiterate, they are not inherently better than other means of representing reality. Furthermore, if measurement is done badly – given its easy transmissibility and institutional and social currency – it has the potential to produce a correspondingly large amount of harm.

### Obstacles, risks and benefits

Our suggestion has workforce planning and training implications. If institutions are to employ people who are skilled in qualitative methods, such staff will need to understand not only relevant practical techniques (coding, data presentation etc.) but standards of – and contests about – qualitative rigour. They will also be networked with academic and research communities, who can support the translation of these forms of expertise. It also involves persuading senior managers and clinicians, including board members, to create space for, and attach salience to, qualitative data analyses alongside metrics in day-to-day meetings and other processes. This is not an easy task, especially in strongly medicalized contexts, where there has been a longstanding contention about the contribution of qualitative methods.^40^

Large-scale adoption of this approach would improve the quality of measurement in health services and would, over time, strongly broaden and strengthen cultures of knowing in health systems. However, even piecemeal adoption could produce significant benefits. PCOMs provide a relatively clear-cut case. In those instances where patients and/or clinicians are using self-defined qualitative ‘measures’ of outcomes, managers would be greatly advantaged if these could be synthesized and used to complement metrics from standardized patient outcomes measures and clinical metrics. They would enable service planners to both ‘read through’ and see beyond familiar metrics and obtain a rich and textured feel for the social contribution their service is making (and where it might be falling down).

There are some significant risks in incorporating qualitative data analyses, if not produced and read with sufficient rigour. Services will not be adequately sensitized to, or give sufficient prominence to, data that challenges institutional assumptions and practices. Any data – including qualitative data – can be co-opted for internal or external public relations purposes. However, as noted above, ‘soft intelligence’ is already used in health services and can already be deployed in ‘bad’ as well as ‘good’ ways. In Martin et al.’s terms, we are focussing on the use of systematically collected and relatively ‘tame’ forms of qualitative knowledge as opposed to more diffuse, spontaneous and ‘fugitive knowledge’.^30^ In this context, Martin et al. usefully warn of the disruptive potential of qualitative ways of knowing being lost if they are uncritically incorporated into forms of managerialism. One of the strengths of the now substantial qualitative research tradition is that it can combine ‘problem-solving’ and ‘critical’ spirits.

## Conclusion

There is a disruptive and positive potential in combining quantitative and qualitative ways of knowing. It can dislodge complacency, radically open up new lines of questioning and understanding, and enable new practices. This is consistent with Pflueger’s call to move beyond ‘accounting logic’ in health systems and develop ‘skeptical calculative cultures’ including ‘the cultivation of overlapping and even conflicting measures of quality.^1^ (p 178)’

The systematic incorporation of qualitative data analysis into health services is one practical response to Pflueger’s call. It would increase the responsiveness and validity of measurement as a means of knowing about the quality of health care services. Furthermore, it would press home questions about whether services are oriented optimally. Addressing these questions may support health systems to confront problems that would otherwise remain hidden, and in doing so benefit the people they serve.
